# Interdisciplinary development of a standardized introduction to gene drives for lay audiences

**DOI:** 10.1186/s12874-020-01146-0

**Published:** 2020-11-05

**Authors:** Cynthia E. Schairer, Cynthia Triplett, Anna Buchman, Omar S. Akbari, Cinnamon S. Bloss

**Affiliations:** 1 Department of Psychiatry, University of California, San Diego, 9500 Gilman Drive, MC 0811, La Jolla, California, 92093-0811 USA; 2 Department of Family Medicine and Public Health, University of California, San Diego, La Jolla, CA USA; 3 Center for Wireless and Population Health Systems, The Qualcomm Institute of Calit2, University of California, San Diego, La Jolla, CA USA; 4 Section of Cell and Developmental Biology, Division of Biology, University of California, San Diego, La Jolla, CA USA; 5 Tata Institute for Genetics and Society, University of California, San Diego, La Jolla, CA USA

**Keywords:** Community and stakeholder engagement, Public health, Vector control, Science communication, Genetic engineering, Gene drives

## Abstract

**Background:**

While there is wide consensus that the public should be consulted about emerging technology early in development, it is difficult to elicit public opinion about innovations unfamiliar to lay audiences. We sought public input on a program of research on genetic engineering to control mosquito vectors of disease that is led by scientists at the University of California and funded by the U.S. Defense Advanced Research Projects Agency (DARPA). In preparation for this effort, we developed a series of narrated slideshows to prompt responses to the development of gene drive mosquito control strategies among lay people. We describe the development and content of these slideshows and evaluate their ability to elicit discussions among focus group participants.

**Methods:**

In developing these materials, we used an iterative process involving input from experts in molecular genetics and vector control. Topics were chosen for their relevance to the goals of the scientists leading the program of research. Significant time was devoted to crafting explanations that would be accessible to uninitiated members of the public but still represent the science accurately. Through qualitative analysis of focus group discussions prompted by the slideshows, we evaluated the success of these slideshows in imparting clear technical information sufficient to inform lay discussion.

**Results:**

The collaboration resulted in a series of four narrated slideshows that were used to anchor discussions in online focus groups. Many participants described the slideshows as interesting and informative, while also raising concerns and possible risks that were not directly addressed in the material presented. Open-ended comments from participants suggest that the slideshows inspired critical questions, reflection, and conversation about genetically engineered and gene drive mosquitoes. After the final and most technically complex slideshow, however, some respondents made comments suggestive of overwhelm or confusion.

**Conclusion:**

Our narrated slideshows prompted engaged conversations about genetically engineered mosquitoes among members of the public who were generally naïve to this technology. Narrated slideshows may serve as viable and useful tools for future public engagement on other controversial emerging medical and public health technologies.

**Supplementary Information:**

The online version contains supplementary material available at 10.1186/s12874-020-01146-0.

## Background

### Overview

Given the increasing global impact of vector borne diseases like malaria and dengue, scientists and vector control professionals are working to replace extant methods such as pesticides with novel vector control methods involving the biological modification of mosquitoes and other vectors. Vector control using genetic modification, in particular, may have the potential to reduce costs, work more effectively, and avoid the harmful effects of chemicals used in pesticides [1]. However, the release of genetically engineered (GE) animals is unavoidably political and ethically fraught as any outcome could have unpredictable and, theoretically, far-reaching ecological consequences. Furthermore, tools for national and global science governance and regulation are woefully underpowered to adjudicate the many competing interests and concerns that surround these technologies. Determining how to develop GE organisms for vector control responsibly, safely, and ethically requires information and input from outside the conventional domains of science, as well as communication across many sectors of society. For these reasons, many have emphasized the importance of community and stakeholder engagement (CSE) early on as central to sorting out how we, as a society, should proceed with these new public health tools [2–9].

CSE is increasingly in demand in science, technology, and medicine, especially when technical achievements are likely to have far reaching impacts on society. Commentators have argued that scientists ought to attend to and consider how their work reflects or challenges the diversity of values held within society [8, 10, 11]. While there are many methods for doing this, many of them start by engaging members of the public in discussions of emerging technologies. These discussions require a baseline knowledge of and vocabulary for often complex and little-known topics. In this article, we describe our efforts to create a standardized introduction to GE mosquito control systems to use in the context of online focus groups with lay people. We also present a qualitative analysis of focus group discussions to evaluate how effective these materials were at fostering discussion and diverse points of view on the topic. The content of our materials as well as the methods of development presented here will serve as a resource for others seeking to engage communities and stakeholders on future technological advances in medicine and public health.

### Technical background

Methods that use GE for vector control are developing rapidly with large scale investments by the Bill and Melinda Gates Foundation, Open Philanthropy Project, Wellcome Trust, Tata Trust, the United States (U.S.) Defense Advanced Research Projects Agency (DARPA), Foundation for the National Institutes of Health (FNIH), and other organizations. Some of the most promising new methods under development use GE to create animals that, when released, modify wild populations in a variety of ways. When we were developing our materials, the most well-developed of these systems was Oxitec’s OX513A mosquito. The OX513A is engineered to pass down a tetracycline dependence in the larval stage. When OX513A males are continuously released into wild populations these males seek out and mate with wild females. Consequently, eggs from females that have mated with OX513A males will hatch but will not survive to grow into adult mosquitoes, thus reducing the total number of biting mosquitoes in the area. One limitation of exposing mosquitoes to the broad-spectrum antibiotic tetracycline, however, is that it reduces the fitness of the OX513A males [12, 13]. To overcome this limitation, Kandul and colleagues have proposed precision guided sterile insect technique (pgSIT). Similar to traditional sterile insect technique (SIT) where radiation is used to produce sterile insects, pgSIT introduces sterile males to the environment to mate with wild mosquitoes resulting in non-viable eggs and reducing the overall population. Unlike traditional SIT, however, pgSIT uses GE and laboratory breeding to produce mosquito eggs that, when hydrated, will only hatch sterile male and intersex mosquitoes [14]. Not only does the pgSIT system not require antibiotics, it enables the release of eggs as opposed to adult mosquitoes and therefore does not require mosquitoes to be reared and mechanically sex-sorted at release sites. Another set of proposals involve a GE approach, known as *gene drive*, that would introduce new genetic traits with preferential inheritance into a wild population. Gene drive could be used to introduce lethal genes that could theoretically eliminate an entire wild population over time. Gene drive also could potentially be used to introduce a genetic resistance to disease-causing parasites, like the ones that cause malaria [15, 16], or introduce genetic modifications that would reverse pesticide resistance [17].

### Project background

In 2017, DARPA funded the Safe Genes program [18], which aims to gain a fundamental understanding of how gene editing technologies function and devise means to develop safe and effective strategies for using these approaches for beneficial ends, including public health and medical applications. As part of Safe Genes, DARPA also funded activities aimed at elucidating issues of Legal, Ethical, Environmental, Dual-Use, and Responsible (LEEDR) innovation associated with the technologies being developed. One of the authors (OA) is the Principal Investigator of one team funded under DARPA Safe Genes, specifically, Team California Safe Gene Drives, known colloquially as “Team California.” The overarching aim of the Team California effort is to safely engineer various classes of gene drives to control the *Aedes aegypti* mosquito vector (*Ae. aegypti)*, which can transmit dengue, Zika, and other diseases. While novel mosquito control techniques are especially needed in regions of the world where dengue and Zika are being actively transmitted, California also stands to benefit from the proposed technologies given the presence of the invasive *Ae. aegypti* vector in at least 12 counties [19]. Over the course of the project, Team California has involved eight different laboratories across University of California campuses. The LEEDR component of Team California (led by CB) is made up of social scientists with expertise in psychology, sociology, public health, and science communication. This group was tasked with evaluating the response of California residents to the Team California program of research, including the gene drives being developed.

### Methodological background

This article outlines the process of developing materials for online, synchronous, chat-based focus groups, describes the final form, and evaluates the materials based on feedback from online focus group participants. The focus groups were specifically aimed at collecting information from California residents about a) the perceived acceptability of the gene drive systems being developed by Team California and b) whether there are specific laboratory experiments or design criteria that could be added to the Team California research plan that would address concerns expressed by Californians. Focus groups were comprised of California residents recruited from a national probability-based online panel [20, 21]. From a public health standpoint, understanding Californians’ responses to gene drives for vector control is important because it is possible that these technologies may one day be used in California. Moreover, California has been visible and influential in terms of setting environmental regulatory and governance standards [22, 23]. Therefore, insights gained from engagement work in this region may help inform future research and possible use of these technologies in other parts of the world.

Because we hoped to collect perspectives from a diverse array of Californians living in areas affected and not affected by *Ae. aegypti*, we elected to use online focus groups to eliminate the need for travel and reduce logistical barriers to participation. Furthermore, the original DARPA contract only supported work consistent with a non-human subjects program evaluation. Although anonymity of participants is not a requirement of program evaluations [24], we sought to maintain a high level of privacy for our respondents given that this was not considered human subjects research. Therefore, we elected to conduct our online focus groups using text-chat instead of video.

These focus groups had to include a substantial educational component because few members of the general public are aware of the proposed gene editing technologies and how they will work. Traditionally, in-person focus groups have been convened to record talk and interactions among a group of people on a topic already familiar to them [25, 26]. While GE for vector control has received some media attention, reports have not been frequent enough or of sufficient general interest to be considered common knowledge. Therefore, a primary challenge in collecting public responses to these techniques was presenting accessible and reasonably unbiased information about a rapidly emerging technology in a new field where there is still technical disagreement among experts [27, 28].

Since focus groups first emerged as a legitimate methodology in the social sciences, stimulus materials have been used to generate discussion, first taking the form of war propaganda [29], and now including product prototypes, story boards, mock-up advertisements, concept boards, and videos [30]. Despite the many handbooks on focus group methods, however, relatively little has been published on how to develop stimulus materials. In the most relevant previous work, studies of attitudes toward different emerging technologies have used carefully compiled concept boards to present different framings to in-person focus groups [25, 31–33]. Concept boards have included, for example, images, headlines, and magazine articles chosen to present contrasting viewpoints so as not to preclude participants from expressing alternative interpretations of the technology that may depart from established, official, or pre-conceived ideas about the meaning of the technology. This previous work demonstrates how concept boards can be used dynamically by an in-person moderator to start discussions and prompt group exploration of issues surrounding emerging technology by following the questions raised and the discussions that emerge between participants.

In light of these factors, adopting concept boards to the online focus group format presented two challenges for our work. First, because it relies on a responsive presentation by the moderator, the concept board technique is more difficult to execute in an online, text-based interaction. Any aural component of the presentation would need to be prerecorded and would therefore not be responsive to participant feedback. Second, Team California scientists desired feedback from the public with regard to efficiency, number of repeated releases, and specific control measures associated with different gene drive systems. Our charge to investigate public responses to these technical features made it difficult to use existing framings of the material. To mimic the concept board approach in our format, we would need audiovisual material produced for a general audience that also presented clear and well-informed points of view on the issue. Furthermore, the specific technical features of GE mosquitoes we needed to talk about had not yet been authoritatively framed in public discourse. The concept of gene drive for pest control has been both championed and opposed in the written media [34–39] as well as in some specialized venues like the online forums created by the Secretariat of the United Nations Convention on Biodiversity [40], but these specific features are less likely to be covered even in these written sources. Audiovisual sources were far less specific. After reviewing interviews, news clips, and other videos available on the internet [41–52], we were unable to find existing sources that presented features and control mechanisms clearly, succinctly, and with sufficient accuracy and detail.

Without existing sources to showcase contrasting points of view, we had no choice but to produce our own materials about technical tools and features so new that very little commentary existed about the most socially salient risks and advantages. We endeavored to move beyond the “deficit model” of science communication [53–55] - which assumes that people resist science and technology because they lack technical understanding – and design an introduction to gene drives that would spark conversation about the possible experience of the intervention without requiring intellectual mastery. We hoped that the focus groups would give our team access to fresh points of view, but also recognized that any presentation originating from our team would carry some bias. In the end, we chose to focus on the details of the proposed systems and their likely enactment, leaving it to the participants to tell us about their hopes and their concerns.

We elected to use a series of narrated slideshows to present technical information and to establish focus and common vocabulary across the groups. This strategy took advantage of the online platform we used (Ipsos, formerly GfK, in partnership with FocusVision) that allowed for video, images, polling questions, and text chat to be presented to participants. Narrated slideshows have been found to be as effective as professionally-developed video productions at imparting information to audience members in the context of educational materials for informed consent procedures [56]. Narrated slideshows also provide an opportunity to impart information through images, text, and voice that may otherwise be difficult given the text-based format of the groups. Given that respondents used either their personal computers to participate or computer equipment provided by FocusVision, the narrated slideshows presented in video format also mimic the now common experience of watching internet videos.

Importantly, generating an accessible and accurate presentation of the relevant technological features of gene drives required collaboration of an interdisciplinary workgroup that included investigators, postdoctoral scholars, doctoral students, and research staff across the genetics and social science laboratories that comprise Team California. Here, we describe the process of developing the narrated slideshows, the content of the slideshows, and analyze evaluative comments from focus group participants regarding the slideshows and their content.

## Methods

### Development of slideshows

Background research that included information from several sources informed the content of the slideshows. Specifically, we drew on many University of California, San Diego lectures and seminars organized to showcase emerging work on gene editing and gene drives, as well as Safe Genes and Team California technical meetings that were focused on DARPA-funded work where members of the genetics labs presented their progress. We also conducted a review of the existing literature on current community and stakeholder engagement efforts related to novel vector control [9, 57], as well as meetings with 17 key informants. Key informants included scientists, managers of vector control districts, and experienced regulators. Meetings with key informants provided additional background, such as information about these individuals’ concerns and priorities regarding vector control and gene drives.

**Fig. 1:**
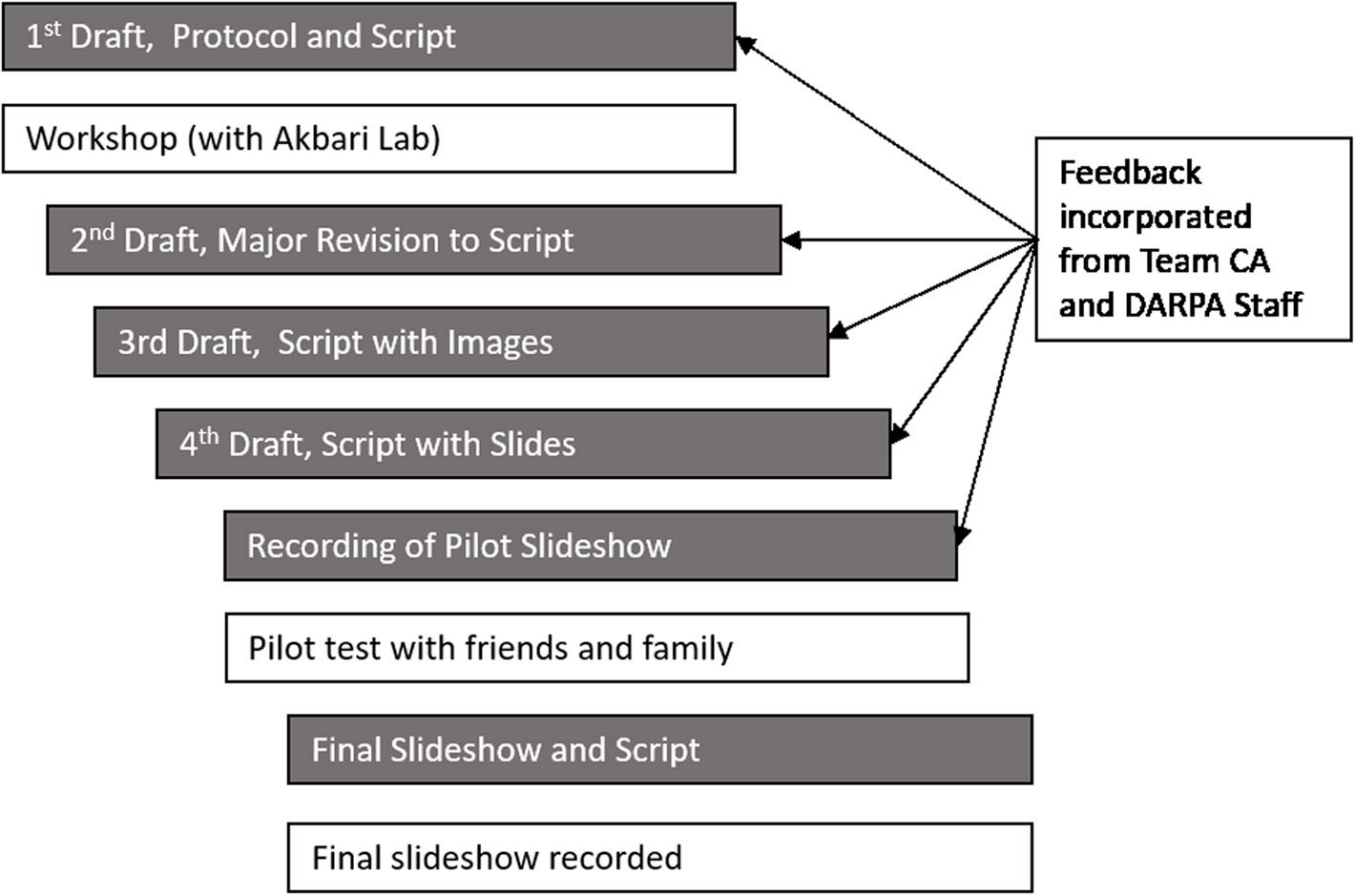
Iterative collaborative process of slideshow development

The initial scripts for the slide shows were developed primarily through an intensive, interdisciplinary, collaborative co-design process involving a workgroup comprised of Bloss and Akbari Lab members, illustrated in Fig. 1. Later drafts were reviewed, revised, and refined based on additional input from other Team California investigators, as well as input from DARPA Safe Genes program staff. As part of the collaborative co-design process, members of the Bloss Lab attended weekly Akbari Lab meetings for nearly a year, co-located with the Akbari Lab 1 day per week; and interacted with Akbari Lab members multiple times per week (in addition to lab meetings) during the 4-month period of active slideshow material development.

Overall, the slideshow went through five major iterations, including revised versions of scripts, slides, and completed videos. Workgroup members collaborated through in-person discussions, phone meetings, commenting on drafts, and more general electronic communication and feedback. The first draft of the focus group protocol (developed exclusively by members of the Bloss Lab) sought to incorporate some of the concerns and questions that arose from the background research and interviews with key informants described above. When reviewed by members of the Akbari Lab and DARPA program staff, however, this initial version was perceived as excluding important technical details relevant to the gene drive systems being developed by Team California, thus prompting the formation of an interdisciplinary workgroup comprised of Bloss and Akbari Lab members. Members of this group worked together on subsequent drafts in an effort to ensure that the script adequately addressed the goals of the Team California LEEDR project, including the technical content and scope of the slideshow materials, and reflected the broader research programmatic content of Team California. The final script was reviewed and iteratively revised based on feedback from OA and other molecular genetics experts within Team California.

**Fig. 2:**
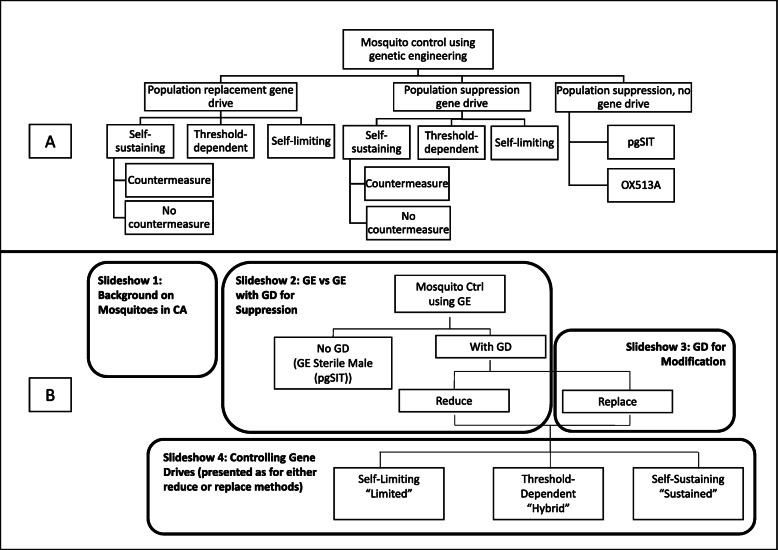
Comparison of schematics of GE vector control technologies developed collaboratively by social science and genetics team members. The original (**a**) was revised and refined to represent the content of the slideshows (**b**)

The feedback process continually clarified the science, including where the science had been misunderstood in previous iterations, and identified additional topics to be covered. At one point during this process, we held a ~ 2-h workshop with eight Akbari Lab members and two Bloss Lab members to discuss these issues. An outgrowth of this process was a collectively-developed schematic of vector control techniques based on gene editing (see Fig. 2a). This schematic captured the details on which the geneticists requested feedback and guided the subsequent slideshow versions. Figure 2 includes both schematics to show how the original schematic (A) was simplified and reorganized to illustrate the final content and flow of the slideshows (B). The slideshows became progressively more complex, moving from comparison of GE sterile males and GE mosquitos with gene drive to comparison of different types of gene drive systems. By doing so, we hoped to collect responses to simpler concepts or comparisons before potentially confusing participants by introducing too much complexity.

Once the script was finalized, existing slides were revised and new slides were developed, primarily by members of the Bloss Lab, though Akbari Lab members provided sample slides and images that required more technical knowledge to construct, such as illustrations of specialized gene drives. CS recorded a set of pilot slideshow videos that were shared with Team California labs and DARPA leadership for final comments. This set of videos was also pilot tested with friends and family. Based on feedback from this piloting process, the content was edited and reorganized, and animations and color coding for important concepts were added. After feedback was incorporated from all reviewers, final versions of the slideshow videos were recorded.

Throughout this process, significant time was devoted to crafting explanations that would be accessible to uninitiated members of the public but still represent the science accurately. To make the information more accessible, we used several strategies. First, we endeavored to select and use words from among the 3000 most common English words [58]. Second, we aimed to describe not only the science behind gene editing and different gene drive systems, but also the outcomes community members would be likely to experience with different approaches. Third, we were attentive to being consistent in our wording across concepts and the need to present no more than two to three concepts at a time followed by soliciting feedback. Fourth, although not ultimately incorporated, we also explored the use of different lay-term metaphors for gene drives (e.g., “tortoise” versus “hare” drives to refer to self-limiting versus self-sustaining gene drives). Piloting the materials with uninitiated friends and family helped us to improve the language, wording, and descriptions, and to correct confusing or misleading images and narration.

### Evaluation of slideshows

To evaluate the effectiveness of these slideshows, we present comments from focus group participants that reflect their comprehension and level of engagement with the content. The slideshows were used to introduce novel mosquito control concepts in 13 English-language, 90-min, online chat-based focus groups conducted between December 2018 and April 2019. Participants were recruited by Ipsos from a national probability-based online panel [20] in four cohorts according to level of education (no bachelor’s degree and bachelor’s degree or higher) and absence or presence of reported *Ae. aegypti* in their county. We also translated these slideshows for use in Spanish-language online chat-based focus groups [59].

The text chat discussions were prompted by polls and open-ended questions. (Additional file 1 provides the moderator guide which includes the questions and their order.) Following each slideshow, participants had time to ask questions and raise concerns with the moderator. Here we analyze evaluative statements that help us understand how effective the slideshows were at fostering meaningful discourse among participants and with the moderator. Quotes from text chat have been edited for grammar and spelling, but not content. Results from the focus groups concerning attitudes toward GE mosquitoes will be reported elsewhere.

## Results

The four slideshows cover 1) mosquitoes in California and basic mosquito facts (Additional file 2); 2) a comparison of GE sterile male mosquitoes with GE mosquitoes with gene drive; (Additional file 3) 3) a comparison of gene drive mosquitoes designed to reduce or suppress populations versus gene drive mosquitoes designed to modify populations (Additional file 4); and 4) a comparison of control strategies for gene drive mosquitoes (self-limiting, threshold-dependent, and self-sustaining with callback measure) (Additional file 5). The topics included, the total number of slides, the duration of each video, and the number of forced choice polling questions are presented in Table 1.

**Table 1:** Structure of Chat-Based Focus Group Sessions

Sequence	Title / Theme	Slideshow Duration	Number of Slides	Forced Choice Polling Questions	Open Discussion Prompts
Opening	Initial Perceptions of the Problem	–	1	2	3
Slide Show 1	“Mosquitoes in California”	*5:10 min*	10	3	2
Slide Show 2	“Genetic Engineering for Mosquito Control”	*5:50 min*	8	4	2
Slide Show 3	“Modifying Mosquitoes with Gene Drive”	*2:49 min*	5	2	1
Slide Show 4	“Controlling Gene Drives”	*5:49 min*	8	4	2
Closing	Review and Discussion	–	–	4	3

### Slideshow 1

The first slideshow, titled “Mosquitoes in California,” briefly discusses the long history of mosquito control in California and the number of mosquito species in the world compared to those in California. It also includes facts about mosquito disease transmission, the mosquito life cycle, and emphasizes that only female mosquitoes bite. The slideshow goes on to explain concerns of public health officials about *Ae. aegypti* mosquitoes. The slideshow explains some of the difficulties of controlling *Ae. aegypti* related to their distinctive habits and preference for biting humans. This slideshow was meant to provide justification for the focus group discussion by explaining to participants why the topic is relevant to California residents.

We knew from our discussions with mosquito control professionals (as well as personal experience talking with acquaintances and family) that most people outside expert circles are not aware of basic facts about mosquitoes that are crucial for understanding the technologies presented in subsequent slideshows. For example, it is impossible to understand why gene editing strategies for mosquito control focus on releasing male mosquitoes unless one understands that only females bite. Likewise, without an appreciation of the differences between *Ae. aegypti* and other common mosquitoes, it is difficult to understand why professionals see this particular mosquito species, which is invasive to California, as a particular threat.

Responses from participants suggest that much of the information about mosquitoes was indeed new to our audience. When the moderator asked, “What did you find most surprising or noteworthy in these slides?” participants reflected back facts from the slideshows. The most common topics mentioned as surprising or noteworthy were the 1) the unique challenge of controlling *Ae. aegypti*, 2) the fact that only female mosquitoes bite, and 3) the fact that *Ae. aegypti* eggs can dry out and remain viable for over a year.

The clarifying questions posed by participants also gave clues about the level of engagement and comprehension. For example, a few participants asked about how to tell the difference between male and female mosquitoes or questioned how this information could be useful, demonstrating not only comprehension, but also that they were challenging or trying to anticipate why this information was highlighted in the slideshow. Other questions also suggested that participants were thinking about possible solutions to these problems. For example, “does a bird bath become dangerous?” (558) and “How big of a problem do we have?” (550).

In response to the forced choice polling question, “Do you agree that public health officials should be worried about *Ae. aegypti* mosquitoes?” 102 of 107 (95%) participants selected “Yes.” These responses, taken together with the comments that discussed the challenge of controlling *Ae. aegypti*, suggest that Slideshow 1 presented an effective argument for why new mosquito control methods are necessary for handling *Ae. aegypti,* thereby setting the stage for the following slideshows. Moreover, in all the analyses presented here, we found no clear differences between cohorts (defined by education and local presence of *Ae. aegypti*.)

### Slideshow 2

Slideshows 2 through 4 each address gene drive systems, including specific features of each system about which Team California molecular genetics labs were most interested in receiving public feedback. As previously described, the choice of these topics and how to structure the presentation evolved through many discussions among interdisciplinary workgroup members. Each of slideshows 2 through 4 were conceived of as reflecting decision points that the geneticists themselves struggle with: to pursue GE techniques with or without gene drive; to use gene drives for population suppression or replacement; and to pursue self-sustaining gene drives or develop less efficient, but more tightly controlled systems. These decision points were elucidated in our early schematic of GE vector control technologies (Fig. 2) and guided the structure and development of the slideshows. Notably, we tried several different ways of organizing the presentation to avoid overwhelming or confusing our audience. Participant responses to the final product suggest that we largely succeeded in making most of the information digestible.

Slideshow 2, “Genetic Engineering for Mosquito Control” presents and compares two strategies for mosquito control that use GE. In all the slideshows, we aimed to describe what community members might experience should local authorities choose to use various GE mosquito control approaches. The first instance of this appears in the narration for the second slide of Slideshow 2, which presented a generic scenario that could describe most GE mosquito systems:

*What would it be like if your community used genetic engineering to reduce the number of* Ae*.* aegypti *mosquitoes in your region? First, you might notice local authorities releasing male mosquitoes from trucks or drones once in a while. There would be more mosquitoes after these releases, but you wouldn’t be getting bitten more (remember, male mosquitoes do not bite). Some time later, you might begin to notice fewer and fewer day-biting mosquitoes in your neighborhood. There would be no need for local authorities to spray pesticides to reduce the number of Ae. aegypti mosquitoes, but it would be necessary for them to release many genetically engineered male mosquitoes. This is what you might experience, but how would this work?*

**Fig. 3:**
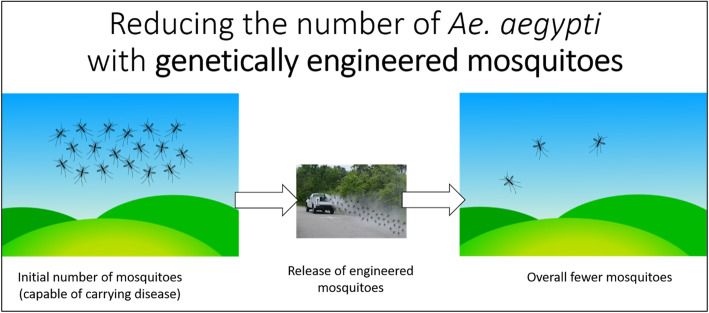
Illustration of community experience of GE techniques from Slideshow 2. The figure was compiled by the team using images in the public domain or under Creative Commons license.(Photography credit: U.S. Air Force photo by Staff Sgt. Teresa J. Cleveland, public domain, mosquito silhouettes added by authors (https://www.jble.af.mil/News/Photos/igphoto/2001551198); Cartoon Grass and Sky: Copyright Studio Freya, mosquito silhouettes added by authors, Attribution-ShareAlike 4.0 International License (https://creativecommons.org/licenses/by-sa/4.0/#))

This description, paired with the image in Fig. 3, was carefully written to help participants visualize how the technologies would work and how participants might experience the impacts of different approaches. This was done prior to presenting the more technical details about how each system would work, which included descriptions and comparison of GE sterile male systems and GE mosquitoes with a population suppression gene drive.

The first system presented was a GE sterile male system, loosely modeled on Oxitec’s OX513A mosquito and the pgSIT system under development in the Akbari Lab [14]. The system described in the slideshows would release male mosquitoes engineered to be sterile to mate with wild female mosquitoes, resulting in fewer viable eggs in the population overall. The slideshows emphasized the need for repeated releases over time to keep a local mosquito population small.

**Fig. 4:**
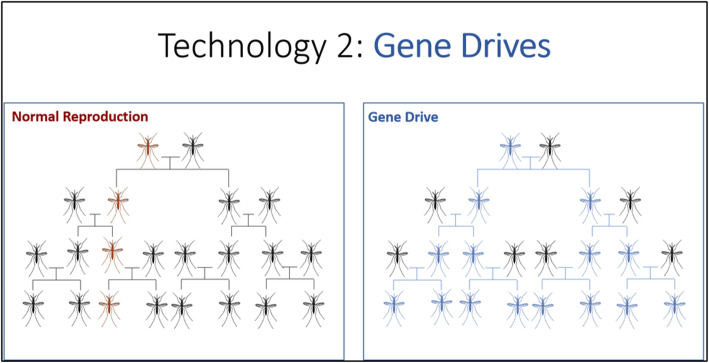
Illustration of gene drive from Slideshow 2

The second strategy presented in Slideshow 2 was GE mosquitoes with gene drive designed to reduce or perhaps eliminate *Ae. aegypti* mosquitoes. The general concept of gene drives was explained with an image (Fig. 4) and the following narration:

*The second technology scientists could use to control Ae. aegypti with genetic engineering would involve a special kind of genetic engineering called “gene drives.” Gene drives make it more likely that an engineered gene will get passed down to an animal’s offspring. In normal reproduction, about half the offspring will inherit a given trait from one parent and half the offspring will inherit that trait from the other parent. In contrast, a gene drive ensures that a particular gene from one parent makes it into most or all the offspring. This means that in normal reproduction, if one parent has the brown gene* [referring to color in image]*, only half of the resulting offspring will inherit that brown gene. Over many generations, only a small number of the animals will have the brown gene. With gene drive, all of the resulting offspring will have the blue gene [referring to color in image]. Over many generations, most of the animals will have the blue gene.*

As an example, we describe a gene drive system of “male mosquitoes that would only ever have male offspring” so that “in each generation, there would be fewer and fewer female mosquitoes to lay eggs.” To emphasize the difference between this technique and GE sterile males, the slideshow proposed a gene drive system that would only require one release to eliminate a population of *Ae. aegypti*.

When participants were polled about previous knowledge of these two techniques, 46 participants (43%) claimed prior familiarity with the GE methods for vector control (selecting “yes” to the polling question, “Before this presentation, had you ever heard about using genetic engineering to control mosquitoes?”) but only 16 participants (15%) claimed prior familiarity with gene drive methods specifically (selecting “yes” to the polling question, “Before this presentation, had you ever heard about mosquitoes with gene drive before?”). Three participants voiced a desire to better understand how gene drive works: “I got GE modification, but not quite sure I understand gene drive” (752), “my first reaction ‘how does a “gene drive” work?’” (117), and “I don’t feel I understand the gene drive as well [as sterile males]” (522). Though one participant commented, “the slides seemed to lead us to support option 2 [gene drive mosquitoes]” (130), subsequent comments and polls demonstrated a mix of responses suggesting that many participants did not accept gene drive mosquitoes as an obvious choice, instead thinking critically about the desirability of gene drive systems. While some participants were positive about the promise of an effective and cost-efficient solution, others maintained throughout their sessions that sterile males were preferable to any gene drive option.

Despite the unfamiliar topic, participants demonstrated the ability to engage with the information, ask many meaningful and relevant questions, and voice concerns and criticism. Some participants commented that they found the slideshow interesting or informative. The most common substantive topics discussed following these slideshows included the possibility of unintended consequences of GE mosquitoes, the potential effects on ecosystems, and whether the methods had been tested for safety and efficacy. These three important topics were raised by the participants themselves, as they were not explicitly discussed in the material that was presented as part of Slideshow 2. In addition, many groups discussed, questioned, or clarified how the presented techniques targeted only one species of mosquito. For example,

*726 does it specifically target certain mosquitos?*

*Moderator @726 The concepts are the same, but mosquito genetics vary by individual types, so Aedes aegypti would be different from any other mosquito in the Aedes family.*

*726 thank you ... so it will not drive all mosquitos just specific species?*

*709 Does that mean mosquitos don't cross breed? So, eradicating one type would not effect* [sic] *others. That would leave the ecosystem balanced?*

*718 so other species of mosquitoes will still be around it's just the Aedes aegypti that would be wiped out?*

*711 oh that sounds reasonable*

In this exchange, participants asked for and discussed clarifying information about the technology (“does it specifically target certain mosquitoes?”) and took into account these discussions as they evaluated the method (“oh that sounds reasonable”). These comments suggest that the slideshows provided enough information to spark such a conversation but did not shut down conversation about targeting by making this feature seem too obvious to question.

### Slideshow 3

The third slideshow, “Modifying Mosquitoes with Gene Drives” presents gene drives designed to “modify” a mosquito population with disease resistance (sometimes described as gene drives for replacement). This is described as an alternative to gene drives designed to suppress or eliminate mosquito populations. This short slideshow, like the others before it, describes how outcomes might be experienced by community members, explaining that modified mosquitoes would be released and that after some time there would be about the same number of mosquitoes in the environment, but they would not be able to carry disease. Following this practical description, a diagram similar to the image on the right in Fig. 4 is used to explain how disease resistance could be introduced into the population using gene drive. For consistency, the images in Slideshow 3 mirror those used in Slideshow 2. Each new method is color coded, but the mechanics of gene drive and the community experience of each method are described in similar terms.

Where the comments following Slideshows 1 and 2 seemed primarily directed at the moderator, in seven of 13 total groups, comments following Slideshow 3 became more directed to and from other participants. The alternative of modifying mosquitoes rather than eliminating them appeared to open up deeper conversations about, for example, the importance of maintaining the species, the relevance of their non-native status, and the severity of the public health threat they posed. In some groups, participants debated these points with little input from the moderator. While they would ask the moderator for factual clarification, they expressed agreements and disagreements with their fellow participants. For example:

*130 I also fear for the precedent*

*128 People might change their tune if they or their family members got sick with these horrible diseases*

*130 we are going to take stingers out of bees*

*130 an* [sic] *teeth off of sharks*

*130 where does it stop?*

*120 Yes, people always change their tune when people get diseases.*

*120 @130 if one of your family member got the diseases, I think you'd change your opinion*

*130 and then we go on hysteria drives and then regret having eliminated that species*

*119 Bees and sharks don't carry disease...That's the point of Reduction of mosquitoes.*

*128 Zika babies born with small head and brain with severe brain damage caused by mosquito bites*

*130 I guess it matters how close we are to an epidemic*

This exchange is an example of how the slideshow provided a starting point for a debate about the possible risks and merits of GE mosquitoes and allowed us to observe the issues and sentiments sparked by these technologies.

### Slideshow 4

The fourth slideshow, “Controlling Gene Drives” presents the three main types of gene drive systems currently described in the scientific literature, which are known as self-limiting, threshold dependent, and self-sustaining. The slideshow presents and compares these systems as different strategies to control gene drives systems. This is the most technical of the four slideshows, and is included because the molecular genetics labs viewed these “control strategies” as critical elements on which public input would be useful. To aid audience comprehension, we created icons for, color-coded, and renamed each category.

**Fig. 5:**
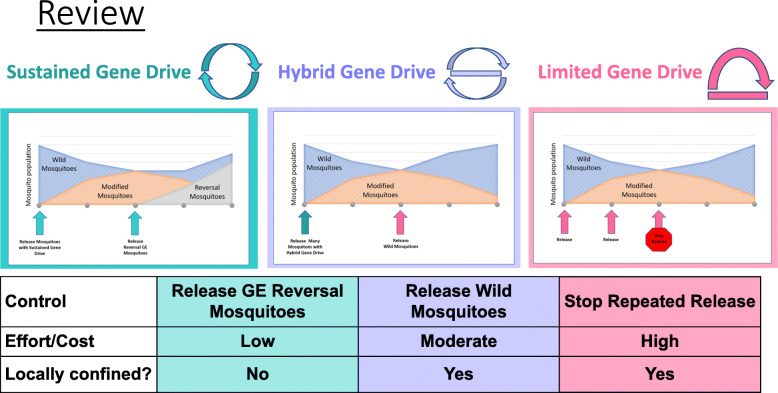
Slide comparing strategies for controlling gene drive systems from Slideshow 4

A few alternative sets of names were considered, including a naming scheme based on the fable “The Tortoise and the Hare,” to emphasize the relative speed and effort required for each. Renaming threshold-dependent drives was particularly difficult due to the complexity of the concept of a threshold. Threshold-dependent drives only work when the right number of GE mosquitoes are released. Under that number, the genes effectively “fall out” of the population after a given number of generations. Comprehension of why a threshold-dependent drive works this way (and why it is called threshold-dependent) requires a level of understanding of population genetics that we did not expect most viewers to gain in the space of a 90-min focus group. Ultimately, the team settled on “sustained gene drives” (for self-sustaining), “hybrid gene drives” (for threshold dependent), and “limited gene drives” (for self-limiting). Threshold-dependent drives became “hybrid drives” to locate them in relation to the other versions of gene drive in terms of cost, human effort, and predicted ability to spread to neighboring populations. Again, each of the three types of drives are presented as different ways to control a gene drive system, as illustrated in the slideshow’s review slide (Fig. 5) and the narration below:

*Scientists have three main ideas for how to control gene drives. Sustained Gene Drives would be controlled by releasing another set of Genetically Engineered Reversal Mosquitoes. Hybrid Gene Drives would be controlled by releasing a set of wild mosquitoes. And Limited Gene Drives would be controlled by stopping repeated releases of gene drive mosquitoes. Let’s take a closer look.*

Following Slideshow 4, the moderator asked each group, “What is your first reaction to the information you just heard?” Some respondents immediately voiced preferences and opinions about the options presented in Slideshow 4, some had specific questions, some sought additional discussion of certain details (e.g., the desirability of geographical confinement), and others expressed general confusion, information overload, or concern.

Compared to the other slideshows, Slideshow 4 prompted more general comments about amount and complexity of the information presented relative to other slideshows. For example, “I think my brain is going to explode” (709) and “I’m having a difficult time following all this information” (501). There were also more specific points of confusion. Because the narrative for Slideshow 4 was vague about why reversal might be desirable, some participants voiced confusion about why anyone would release wild-type mosquitoes or why all the examples showed the mosquito population returning to previous levels. Despite these sources of confusion, many participants were able to discuss the presented information and form opinions about the different options.

Slideshow 4 stimulated conversations that allowed us to observe some participants clearly grappling with the practical, political, and ethical tradeoffs of each type of gene drive. For example, some participants clearly weighed control and cost when commenting, “Being able to confine them locally would be a good control method and make the cost worth it” (109) or “I think that local confinement is a must” (763). Another participant demonstrated comprehension of the utility and cost of a call back measure: “I’m torn. If something did go wrong, if it took a long enough time to notice, the reversal process might be expensive in terms of time & money” (516). This is precisely the type of deliberation we aimed to prompt and observe in this project.

## Discussion

We developed a series of narrated slideshows to facilitate responses and discussion among lay people about a program of research on GE mosquitoes with gene drive. Here we have described the process of developing these slideshows, their content, and feedback from the focus group participants who viewed them. Overall, responses among participants suggest that our stimuli prompted engaged conversations about GE mosquitoes among California residents who were generally naïve to this technology. The primary challenge of the approach was to present highly technical information in a digestible and reasonably unbiased form. Narrated slideshows can serve as viable and useful stimuli for social science efforts endeavoring to prompt and facilitate meaningful lay conversations about emerging technologies.

### Comprehension

In general, responses to the slideshows represented a level of engagement with the topics that would have been impossible without a reasonable level of comprehension. For example, while many participants expressed optimism about the possibilities of these new technologies, many respondents weighed the options, differentiated between techniques, and also expressed ambivalence when asked about the acceptability of gene editing to control mosquitoes. Participant responses suggest that Slideshow 1 effectively set the stage for the discussions by raising awareness of a problem (*Ae. aegypti* mosquitoes) that many participants were not aware of before the group. Slideshow 2 appeared to have provided enough information to encourage questions, while not providing so much as to discourage conversation.

Discussions following Slideshow 3 were qualitatively different than those that followed the first two slideshows. Participants began discussing pros and cons among themselves, comparing and weighing the risks and concerns raised by the group with the potential of the technology to address public health concerns and the nuisance of mosquitoes. This may reflect a moment in the focus group when “the ice was broken”, and participants began to feel more comfortable with the topic. The repetition of gene drive mechanics in Slideshow 3, as well as the contrast presented between reduction and modification of *Ae. aegypti*, may well have clarified the issues for some and contributed to the apparent increased comfort engaging with the subject matter. Although some commented on the large amount of information in Slideshow 4, discussions and debate among the participants continued in many of the groups, allowing us to observe participants comparing and weighing the control mechanisms that were presented.

### Bias

Any account, including narratives of science, cannot be disentangled from the point of view of the author [60]. Therefore, we thought carefully about how to impart needed information about these technologies with a minimum of bias. It may be argued that Slideshow 1 set up participants to accept gene drive technologies by focusing on the concerns about mosquitoes and not address “what the benefits of mosquitoes are” (550), as one participant commented. If the purpose of Slideshow 1 was to stimulate debate about whether mosquitoes are a threat, the argument would have been too strong. However, the need to engage with members of the public about GE mosquitoes does not stem from uncertainty about the threat of mosquitoes so much as from uncertainty about the acceptability of using GE methods as a solution. We felt that, without presenting the motivation for using GE to control mosquitoes in California, the remainder of the focus group would have become a theoretical discussion about a technology too removed from the participants’ interests. Slideshow 1 served to establish the problem and a common vocabulary about that problem, without which GE mosquitoes with gene drive could not have been productively discussed.

In the subsequent slideshows we attempted to present how the technologies would likely be experienced by community members and how they are meant to work. We deliberately kept these slideshows silent about possible risks and unintended consequences to see what the groups would generate on their own. We might have strengthened this strategy by taking more care to present technical features without unnecessarily highlighting their desirability. For instance, the narration in Slideshow 2 did not need to mention that GE techniques would not require pesticides. In Slideshow 4, cost and efficiency may have been over-emphasized while the rationale for control measures may have been understated.

We strived to create materials that would not preclude participants from expressing their opinions and would minimize social desirability bias in the data. Where previous focus group studies on emerging technologies minimized social desirability bias by presenting contrasting viewpoints [25, 31–33, 61], our need to present technical features led us to maintain an educational tone that focused on the potential community experience of the technologies. We note that the emphasis on the threat of *Ae. aegypti* in California in Slideshow 1 did not convince all participants that GE technologies were necessary. Nor did the relative silence in the slideshows on possible unintended consequences preclude these topics from being discussed. Indeed, in every group, participants raised concerns about possible unintended consequences including ecological impact, genetic resistance, or dangerous mutations. (A paper describing these substantive results of the focus groups is in preparation.) In short, while the stimuli generally did not directly address these issues, it appears that they included enough information for participants to draw on their own prior knowledge, interests, and values to provide thoughtful feedback.

### Limitations and lessons

Combined with the strong argument made in Slideshow 1 regarding concerns about *Ae. aegypti*, our emphasis in later slideshows on features likely to be seen as benefits constituted a bias that we only came to appreciate in retrospect. When developing Slideshow 1, we sought to explain the motivation for the research, but we might have opted instead for a more detached animal documentary style in which information was organized around a natural history of *Ae. aegypti* rather than their status as a worrisome public health threat. Regardless of Slideshow 1, removing references to pesticides in Slideshows 2 and 3 and adding more explanation for why containment of gene drive systems may be desirable would have improved these materials.

Due to limitations related to the project’s designation as a program evaluation, we know very little about the participants beyond a binary grouping based on educational level and if their zip code was associated with a county where *Ae. aegypti* had been reported. However, by using a probability sample as the recruitment source for this project, we aimed to minimize unintended and unobserved sources of selection bias. The online format of our groups also limited the types of feedback we could get from participants, since we had no access to non-verbal cues. Therefore, we can only evaluate what participants chose to write into the chat box and their answers to polling questions; we have no information about what may have gone unsaid, especially by people who may have been too unsure to formulate a question or statement, though these latter issues are often at play in in-person focus groups as well. Overall, however, in most of the groups, a majority of participants substantively contributed to the chat conversation.

Similar narrated slideshows could be used in other efforts to collect public responses to emerging technologies in public health and medicine, especially as institutions place more emphasis on patient-centered and community-informed health interventions. Furthermore, this type of slideshow is amenable to diverse formats (i.e., online or in-person) and methods (i.e., interviews, focus groups, or surveys). Future work could explore the relative merits of slideshows and topic boards via systematic comparison. Where topic boards are more dynamic, narrated slideshows may provide the moderator with some distance from the “expert narrative” presented in the slideshow, allowing them to lead a more critical conversation about both the form and content of the stimuli.

## Conclusion

We used an interdisciplinary, collaborative co-design process to develop a series of narrated slideshows to anchor online, chat-based focus groups and facilitate collection of lay evaluation of a gene editing research program. By investing the time into an iterative process involving multiple disciplines and individual experts, we created an example of a standardized introduction to gene drive mosquitoes that prompted sophisticated questions, useful comments, and engaged dialogue from lay participants. In many cases, these slideshows appeared to have presented enough technical detail to inform discussions and opinions, but not so much as to overwhelm or shut down discussions.

In this project, we aimed to listen to how members of the public responded to and made sense of the technical vision that our team was building in the lab and ultimately incorporate what we learned into the technical development. To foster meaningful discussions, we needed to educate only enough to begin a conversation we sought to hear but did not desire to control. This required us to balance accessibility and technical accuracy in our explanations as well as navigate the tension between worries and skepticism about these technologies and the motivations for developing them. This type of engagement between innovators and members of the public is increasingly important in medicine and public health, where the technologies wielded by professionals have far-reaching effects on the lives of many. This detailed description of our process and methods is provided to inform future efforts to foster these very important discussions.


**Additional file 2.**Slide Show 1: Mosquitoes in California


**Additional file 3.** Slide Show 2: Genetic Engineering for Mosquito Control


**Additional file 4.** Slide Show 3: Modifying Mosquitoes with Gene Drives


**Additional file 5.** Slide Show 4: Controlling Gene Drives

## Supplementary information

**Additional file 1.** Focus Group Guide

## Data Availability

The datasets used and/or analyzed during the current study are available from the corresponding author on reasonable request.

## Abbreviations

DARPA: Defense Advanced Research Projects Agency
GE: Genetically engineered
LEEDR: Legal, Ethical, Environmental, Dual-Use, and Responsible innovation
NIH: National Institutes of Health
pgSIT: Precision guided sterile insect technique
SIT: Sterile insect technique
US: United States
