# Supplementary material for: Interdisciplinary development of a standardized introduction to gene drives for lay audiences
Source: BMC Med Res Methodol. 2020 Nov 5;20:273. doi: 10.1186/s12874-020-01146-0 (PMC7643426; doi:10.1186/s12874-020-01146-0)
Supplement: Supplementary file 5 — Additional file 1. Focus Group Guide [file 12874_2020_1146_MOESM1_ESM.docx]

**Interdisciplinary development of a standardized introduction to gene drives for lay audiences**

**Schairer, Triplett, Buchman, Akbari, and Bloss**

**Focus Group Guide**

|  | **Format** | **Image/Video** | **Polling Question** | **Answer Categories** |
| --- | --- | --- | --- | --- |
| 1 | Video of opening remarks | Opening Remarks slide |  |  |
| 2 | Poll & chat | No image | As far as you know, are mosquitoes a problem in your area? | Yes  No |
| **DISCUSSION:** Tell me about your answer. | | | | |
| **DISCUSSION:** If someone has a lot of mosquitoes around their house, what can they do? | | | | |
| 3 | Poll & chat | No image | As far as you know, is there a public agency in your area that deals with mosquitoes? | Yes  No |
| **DISCUSSION:** How do you know? Have you seen them or heard from them yourself? | | | | |
| START VIDEO SLIDESHOW 1 (5:10) | | | | |
| 5 | Text chat | P11 SS1 Review  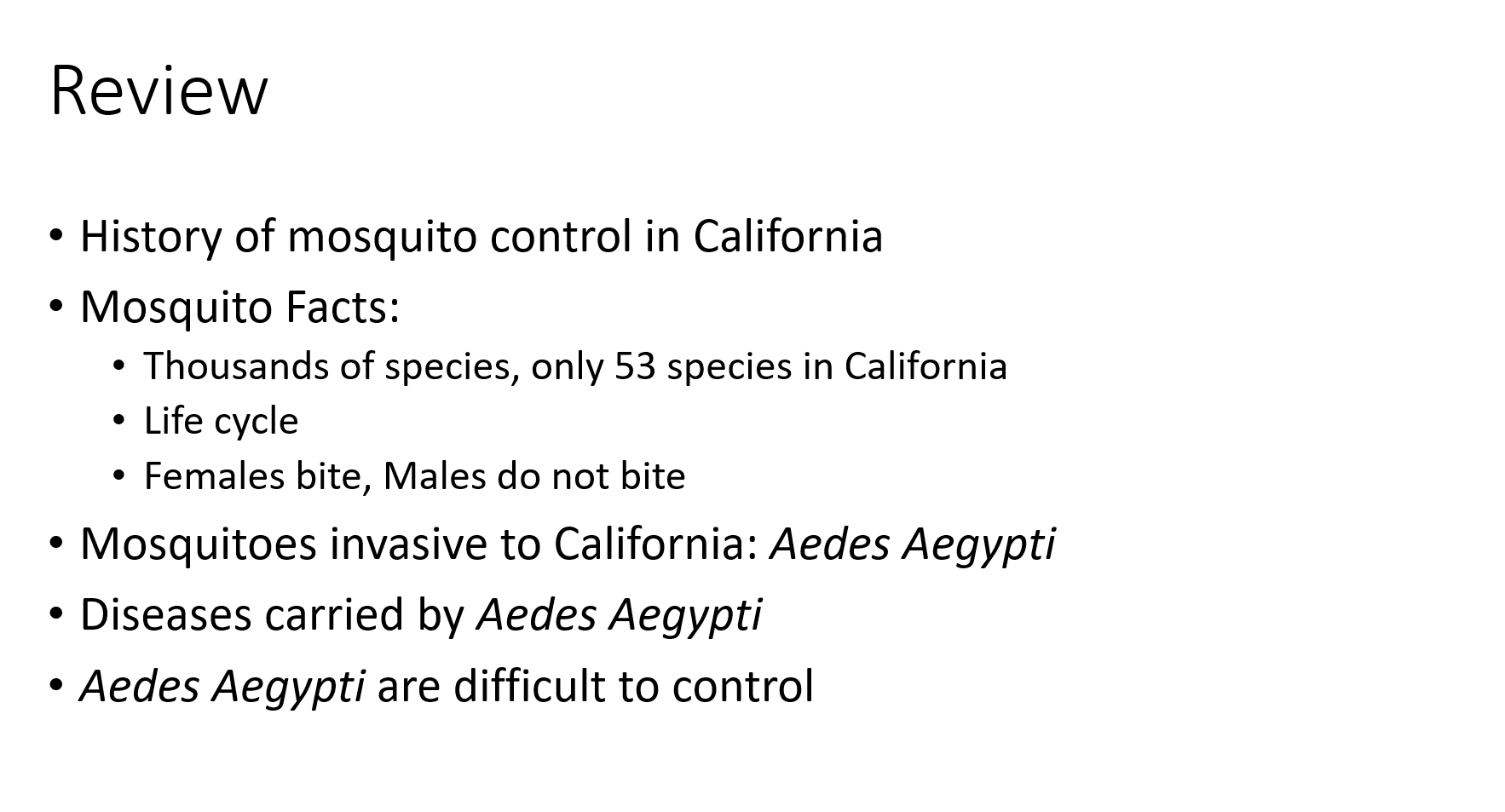 | **DISCUSSION:** What did you find most surprising or noteworthy in these slides? |  |
| 6 | Poll & chat | No image | Before you saw this, had you ever heard about Aedes aegypti mosquitoes before? | Yes  No |
| 7 | Poll & chat | No image | Before you saw this, did you know that disease carrying mosquitoes can be found in some parts of CA? | Yes  No |
| 8 | Poll & chat | No image | Do you agree that public health officials should be worried about Aedes aegypti mosquitoes? | Yes  No |
| **DISCUSSION:** Please tell me about why you answered the last question the way you did. Why do you agree or disagree that officials should be worried about Aedes aegypti mosquitoes? | | | | |
| START VIDEO SLIDESHOW 2 (5:50) | | | | |
| 10 | Poll & chat | No image | Before this presentation, had you ever heard about using genetic engineering to control mosquitoes? | Yes  No |
| 11 | Poll & chat | No image | Before this presentation, had you ever heard about mosquitoes with gene drive before? | Yes  No |
| **DISCUSSION:** What is your first reaction to the information you just heard? Do you have any questions about these slides? | | | | |
| 12 | Poll & chat | P23 GE Sterile Males  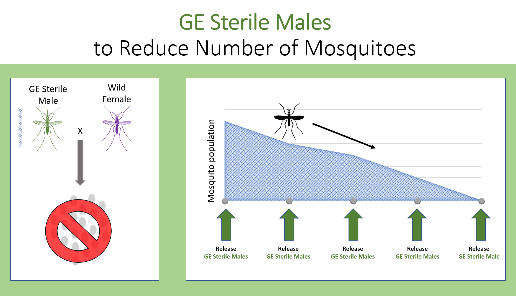 | Based on what you have just heard, do you feel that it would be acceptable to use GE Sterile Males to reduce the number of Ae. aegypti mosquitoes in an area? | Yes  No |
| 13 | Poll & chat | P24 Gene Drive  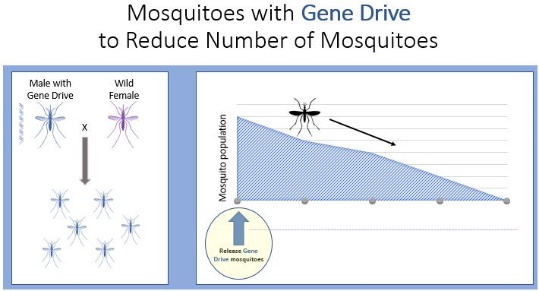 | Based on what you have just heard, do you feel that it would be acceptable to use mosquitoes with Gene Drive to reduce the number of Ae. aegypti mosquitoes in an area? | Yes  No |

| START VIDEO SLIDESHOW 3 (2:49) | | | | |
| --- | --- | --- | --- | --- |
| 15 | Poll & chat | P31 Modify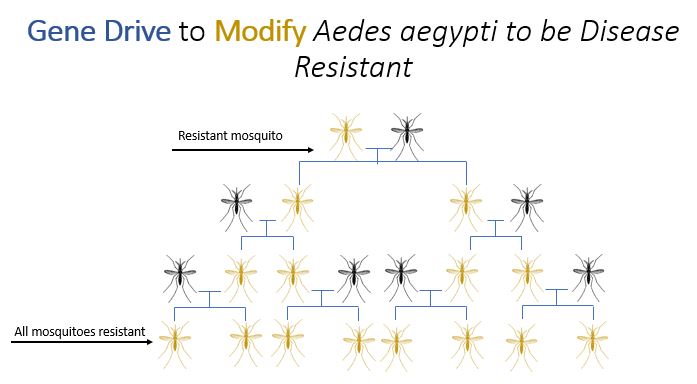 | Do you feel that it would be acceptable to use gene drive to modify mosquito populations? | Yes  No |
| 16 | Poll & chat | P32 Reduce Modify  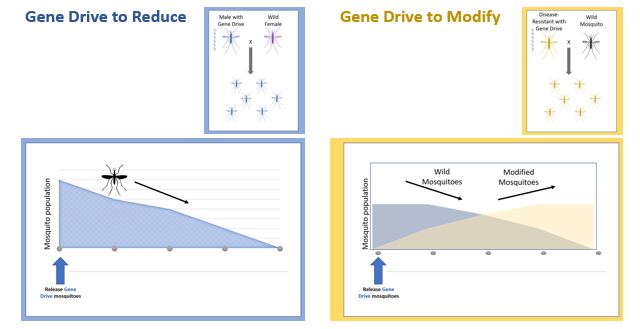 | Which type of mosquito control strategy would you be most likely to support in your community? | 1. GE to **REDUCE** the number of mosquitoes  2. GE to **MODIFY** the number of mosquitoes  3. I would **not support either** strategy  4. I would **support both** strategies |
| **DISCUSSION:** Do you have any worries about these ways of using gene drive to control mosquitoes? | | | | |
| START VIDEO SLIDESHOW 4 (5:49) | | | | |
| 18 | Text chat | P41 Sustained  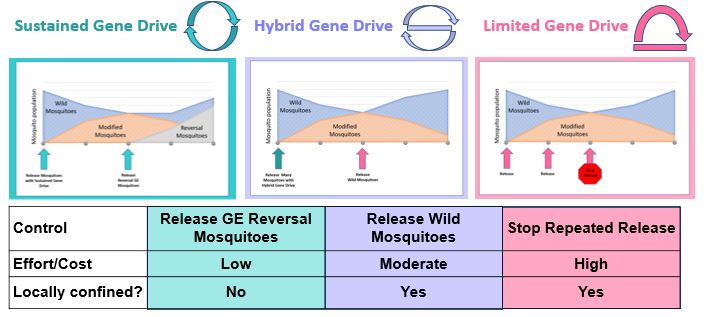 | **DISCUSSION:** What is your first reaction to the information you just heard? Do you have any questions about these slides? |  |
| 19 | Poll & chat | P42 Hybrid  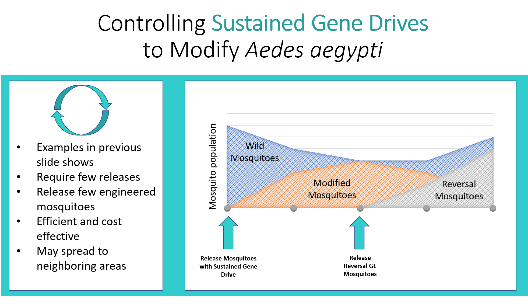 | Would it be acceptable to use Sustained Gene Drives to control mosquitoes? | Yes  No |
| 20 | Poll & chat | P43 Limited  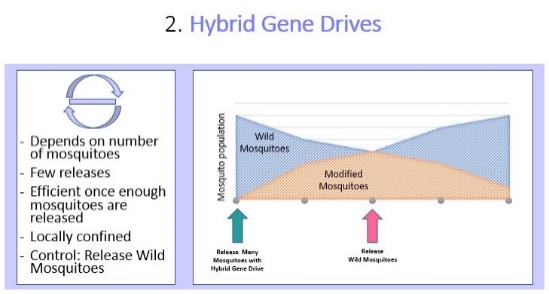 | Would it be acceptable to use Hybrid Gene Drives to control mosquitoes? | Yes  No |
| 21 | Poll & chat | P43 Limited  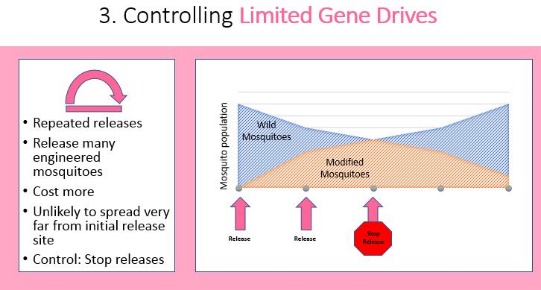 | Would it be acceptable to use Limited Gene Drives to control mosquitoes? | Yes  No |
| 22 | Poll & chat | P44 Control Compare  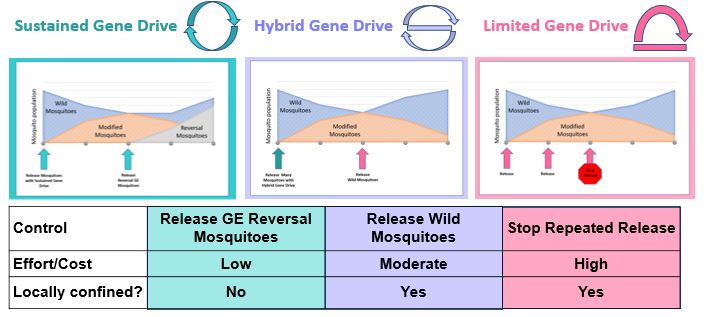 | Thinking about the pros and cons you just learned about, which type of gene drive would be most acceptable to use in your community? | **1. Limited** Gene Drives  **2. Sustained** Gene Drives  **3.Hybrid** Gene Drives |
| **DISCUSSION:** Tell me about your answers to these questions. | | | | |
| 23 | Poll & chat | No image | Now that you have learned about the possibilities of genetic engineering to control mosquitoes, do you think genetic engineering options would be better or worse than using pesticides? | 1. GE would be **BETTER** than pesticides  2. GE would be **WORSE** than pesticides |
| **DISCUSSION:** Please tell me why you chose your answer. | | | | |
| 24 | Poll & chat | No image | In the future, should local mosquito control authorities be able to use any of these methods? | Yes  No  Undecided |
| 25 | Poll & chat | No image | Would you feel comfortable with your community using GE mosquitoes if they were found to be safe by scientists working for the government? | Yes  No  Undecided |
| 26 | Poll & chat | No image | Would you approve of funding research into these kinds of mosquito control strategies with taxpayer money? | Yes  No  Undecided |
| **DISCUSSION:** Tell me about your answers. | | | | |
| **DISCUSSION:** Is there anything else you think I should have asked about? Anything else you want to talk about? | | | | |
